# Towards Equity in Service Provision for Gay Men and Other Men Who Have Sex with Men in Repressive Contexts

**DOI:** 10.1371/journal.pmed.1002154

**Published:** 2016-10-25

**Authors:** Chris Beyrer, Keletso Makofane, Ifeanyi Orazulike, Daouda Diouf, Stefan D. Baral

**Affiliations:** 1 Center for Public Health and Human Rights, Johns Hopkins Bloomberg School of Public Health, Baltimore, Maryland, United States of America; 2 International AIDS Society, Geneva, Switzerland; 3 The Anova Health Institute, Johannesburg, South Africa; 4 International Center for Advocacy on Rights to Health (ICARH), Abuja, Nigeria; 5 Enda Santé, Dakar, Senegal

## Abstract

Chris Beyrer and colleagues reflect on an underappreciated trend in multiple African, Asian, and Caribbean settings, in which the provision of HIV and other essential health services for sexual and gender minorities is expanding despite challenging legal and social environments.

At the 21st International AIDS Conference in July, 2016, in Durban, South Africa, The Elton John AIDS Foundation (EJAF) and the United States President's Emergency Plan for AIDS Relief (PEPFAR) program announced the inaugural awardees of the new US$10 million initiative to increase access to HIV services for lesbian, gay, bisexual, and transgender (LGBT) individuals and communities in Africa and the Caribbean. The awardees of the fund were the International HIV/AIDS Alliance (IHAA) and the Global Forum on Men Who Have Sex with Men (MSM) and HIV (MSMGF), both community-based organizations with track records in providing support at the grassroots to LGBT populations, including in many countries where sexual and gender minorities remain stigmatized and, too often, criminalized for their behaviors, identities, or gender expression [1]. The LGBT Fund, first announced in late 2015 by Sir Elton John and Ambassador Deborah Birx of PEPFAR, was created to address these harsh realities and to expand HIV prevention and treatment access for LGBT persons.

This extraordinarily welcome step speaks to a larger and little-appreciated trend underway in multiple African, Asian, and Caribbean settings, in which the provision of HIV and other essential health services for sexual and gender minorities are expanding despite challenging legal and social environments. Although in some high HIV burden countries there is strong opposition to such efforts, in others—Kenya is an example—coverage and quality of service provision continue to improve despite legal obstacles. The government of Kenya recently approved pre-exposure prophylaxis (PrEP) for gay and other MSM without first decriminalizing same-sex sexual acts [2]. South Africa is the only other African country to approve PrEP for gay men and other MSM.

Also, at AIDS 2016, the International AIDS Society and EJAF launched a new initiative—*Me and My Health Care Provider*—to celebrate physicians, nurses, and other providers offering culturally competent HIV and other sexually transmitted infections (STI) prevention, treatment, and care services for key population patient groups, including gay men, other MSM, and transgender clients [3]. Providers are nominated by their patients, and a number of the provider–patient pairs were present in Durban to launch the campaign. A wonderful example was a young physician from Cameroon, Dr. Hermine Meli from Yaoundé Central Hospital, who was nominated by two of her patients. Yet Cameroon currently criminalizes same-sex practices and has been the scene of harsh homophobic violence, including the killing of Eric Lembembe, a prominent LGBT rights activist, in 2013 [4]. Cameroonian government support for clinicians to provide essential services to MSM highlights the complex dynamics present in many countries where members of health-oriented ministries and local community-based organizations facilitate programs and research for sexual and gender minorities, often at significant personal and professional risk.

These efforts are encouraging and are forging new paths in difficult environments. At the same time, evidence that anti-gay laws negatively affect HIV outcomes continues to mount. For example, the TRUST cohort, a large prospective cohort study of Nigerian MSM living with or at risk for HIV, provided the context for a natural experiment of sorts when, in 2014, Nigeria imposed a harsh anti-gay law, the “Same-Sex Marriage Prohibition Act,” which extended Nigeria’s existing sodomy laws [5]. In addition to documented physical and structural violence precipitated against gay men [6,7], the law has had other consequences for long-term health. Among the TRUST participants who continued their participation, there were measurable increases in the fear of seeking health care and in avoidance of health care visits [8]. Tragically, a substantial proportion of the TRUST cohort, in which HIV prevalence is almost 50% and annual HIV incidence is estimated to be 14%, was lost to follow-up after the imposition of the law. Fears around identifying as MSM may also have consequences for treatment, as men living with HIV in this study were less likely to have achieved viral suppression if they had not disclosed MSM status to their provider [8]. Furthermore, by criminalizing the operation of LGBT organizations, the law threatens programs that deliver lifesaving health services. That these very programs provide crucial advocacy to improve the social and legal environment in Nigeria serves to compound the law’s damages [9].

Other studies from Malawi, Namibia, and Botswana—all countries that criminalize same-sex behavior—have also demonstrated that fear of seeking health care because of sexual minority status is a potent barrier to seeking HIV services, including HIV testing, the first critical step in engaging in either the prevention or treatment continuums of care [10].

Therefore, although improving health services in the face of repressive laws is admirable and beneficial, legal reform—often a slow and contentious process—is necessary in the long term. In his dissent from the 2003 US Supreme Court decision overturning sodomy laws across the US, Justice Antonin Scalia objected that decriminalizing homosexuality and same-sex practices more broadly would constitute significant steps towards full citizenship rights (notably marriage) for LGBT persons [11]. Although later decisions showed the late Justice’s prediction to be correct, more than a decade passed before the courts affirmed as appropriate the rights to which he had objected. More recently in the Caribbean, the Belize Supreme Court, ruling in the case of Caleb Orozco, found Belize’s sodomy law to be unconstitutional, and the government of Belize announced in August, 2016 that it would not appeal the ruling [12]. In a victory for both LGBT rights and HIV care, the decision cited the sodomy law as a barrier to HIV services for gay men and noted that it undermined public health goals for the country. One of us (CB) filed expert testimony in support of Caleb Orozco, which the court cited in the ruling:

Criminalization and stigmatization not only perpetuate systematic discrimination and violence that limit the study of HIV risks for MSM; they also restrict the extent to which health care providers can effectively offer and MSM can safely access health care services that would reduce HIV transmission and treat HIV infection (Sullivan et al., 2012) [13]. Criminalization and stigmatization, therefore, complicate the health needs of MSM and act as severe barriers to individual country and global responses to the HIV epidemic. [14]

While the legal, human rights, and LGBT advocacy communities work to advance structural reforms such as overturning sodomy statutes, community-based clinical and service providers have vital roles to play in the realization of more immediate rights and benefits, including the right to dignity, safety, and equitable access to services in health care settings. The progress that they are making is essential, because the sustained high rates of incident HIV infections among young gay, bisexual, and other MSM, globally as in the US, remain a critical challenge to controlling the HIV epidemic.

## Abbreviations

EJAF: The Elton John AIDS Foundation
IHAA: International HIV/AIDS Alliance
LGBT: lesbian, gay, bisexual, and transgender
MSM: men who have sex with men
MSMGF: Global Forum on MSM and HIV
PEPFAR: President's Emergency Plan for AIDS Relief
PrEP: pre-exposure prophylaxis
STI: sexually transmitted infection
